# Effect of the Duration Time of a Nuclear Accident on Radiological Health Consequences

**DOI:** 10.3390/ijerph110302865

**Published:** 2014-03-10

**Authors:** Hyojoon Jeong, Misun Park, Haesun Jeong, Wontae Hwang, Eunhan Kim, Moonhee Han

**Affiliations:** Nuclear Environment and Safety Division, Korea Atomic Energy Research Institute, 989-111, Daedeok-daero, Yuseong, Daejeon 305-353, Korea; E-Mails: pmisun@kaeri.re.kr (M.P.); haesunin@kaeri.re.kr (H.J.); wthwang@kaeri.re.kr (W.H.); ehkim@kaeri.re.kr (E.K.); mhhan@kaeri.re.kr (M.H.)

**Keywords:** nuclear accident, radiological health consequences, Gaussian plume model, public health, emission characteristics

## Abstract

This study aimed to quantify the effect of duration time of a nuclear accident on the radiation dose of a densely populated area and the resulting acute health effects. In the case of nuclear accidents, the total emissions of radioactive materials can be classified into several categories. Therefore, the release information is very important for the assessment of risk to the public. We confirmed that when the duration time of the emissions are prolonged to 7 hours, the concentrations of radioactive substances in the ambient air are reduced by 50% compared to that when the duration time of emission is one hour. This means that the risk evaluation using only the first wind direction of an accident is very conservative, so it has to be used as a screening level for the risk assessment. Furthermore, it is judged that the proper control of the emission time of a nuclear accident can minimize the health effects on residents.

## 1. Introduction

Since the Fukushima Daiichi Nuclear Power Plant accident that occurred after an earthquake in eastern Japan in 2011, there has been increasing interest in Korea in nuclear accidents. Emission of radioactive substances caused by severe accidents such as the nuclear accident in Japan has been done over days or tens of days. If the duration time of emission increases, radioactive substances are dispersed, rather than being concentrated in a certain area, as the dilution actions are activated and owing to the influence of weather. This study assessed the radiological consequences by estimating the effect of emission duration on the radiation dose and acute health effects on human beings from a hypothetical nuclear accident. 

The methods for a radiological consequence assessment of a radiation accident in a nuclear facility may be classified as follows according to the presence/absence of an accident at the time of assessment: For an accident having already occurred at the time of assessment, we can specify the source term, the weather conditions, and the measurements of radiation activity at the time of the accident. In this case, we can estimate the actual radiation exposure dose and radiation risk using the released source term and meteorological data at the time of the accident and compare them with the existing measurement data. For a nuclear accident that has not occurred yet, it is necessary to hypothesize the source term to be emitted and the weather conditions. As for the release source term, we use the source term taking into account the occurrence probability according to the accident type, or in a worst case, the source term of accidents such as Chernobyl or Fukushima. A hypothesis on the weather conditions at the time of an accident can be quantified through an analysis of the frequency distribution of atmospheric dispersion factors. This can be achieved by expressing the atmospheric dispersion characteristics in the accident area as atmospheric dispersion factors based on meteorological data measured for one year or a few years. 

Since the Fukushima Daiichi Nuclear Power Plant accident in Japan, voluminous research on the atmospheric dispersion and risk assessment of radioactive substances has been reported [1,2]. The representative examples trace the path of radioactive substances using Hysplit or Flexpart which are particle models, and an estimation of the source term [3,4,5]. In addition, the models developed to assess the behaviors of radioactive substances and health effects caused by radioactive substances have been used for a radiological consequence assessment of a nuclear accident. Among those consequence assessment codes, the MACCS2, which is a probabilistic safety assessment code developed by SNL (Sandia National Laboratory) of the USA, and ADDAM, developed by ACEL in Canada, employ the Gaussian Plume Model to analyze atmospheric dispersion of radioactive substances [6,7].

This study quantified the effect of accident duration time on the concentration of atmospheric radioactive substances, radiation dose, and population risk, in the event that any hypothetical accident assuming Fukushima source term occurs in a nuclear power plant in Korea. This study used the Gaussian Plume Model for an analysis of the fate and transport for radioactive substances, and one-year meteorological data measured in 2011 in Gwangju, Korea to analyze meteorological phenomena.

## 2. Material and Methods

### 2.1. The Gaussian Model

The Gaussian Model is one of the most widely used models in investigating the behavioral characteristics of atmospheric pollutants. It is used to estimate the density of the receptor by hypothesizing the atmospheric dispersion with a statistical Gaussian distribution [8]. Supposing *x* as the downwind distance at the site of an accident, *y* as a quadrature component from the downwind distance, and *z* as a vertical component from the downwind distance, the air concentration (C) of radioactive substances is as follows:

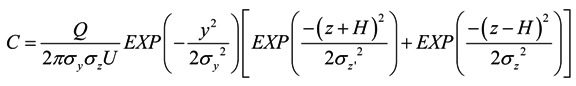
(1)
where Q is a released source term, U is the wind speed, H is an effective plume height and σ_y_ and σ_z_ are horizontal and vertical dispersion coefficients, respectively, which represent the degree of dispersion considering the wind speed, amount of clouds, and day and night conditions. σ_y_ and σ_z_ generally use the empirical formula of Pasquil and Gifford [9].

### 2.2. Estimation of Radiation Dose

The atmospheric dispersion between the release point and receptor points may be characterized by supposing the emission source as an unit mass emission and moving it to the left side. This is called an atmospheric dispersion factor (ADF) and its unit is s/m^3^ [10]. The following shows the relationship among the ground concentration, emission rate, and an atmospheric dispersion factor of radioactive substances:

*C* (*x*, *y*) [*Bqm*^−3^] = *Q* [*Bqm*^−1^]∙*ADF* [(*Bqm*^−3^)] / (*Bqm*^−1^)] (2)

The time integrated air concentration (TIC), in which total quantity (QT) is substituted for the emission rate in the above equation is as follows:

*TIC* (*x*, *y*) [*Bqsm*^−3^] = *QT* [*Bq*]∙*ADF* [*sm*^−3^] (3)

Some of the atmospheric radioactive substances are removed by deposition. The ratio of deposition is decided according to the kind of radioactive substance. As for noble gases, they have a rare effect of deposition, and the deposition varies according to the size and form of the particles. The deposition amount can be expressed as follows:

*DEP* (*x*, *y*) [*Bqm*^−2^] = *v_d_* [*ms*^−1^]∙*TIC* [*Bqsm*^−3^] (4)

Here, *v_d_* means the deposition velocity and 0.1 cm/sec is applied in this study. The radiation exposure dose that a person suffers from the released radiation source consists of internal radiation exposure by inhalation, external radiation exposure by a radiation plume (cloudshine), and external radiation exposure generated from deposited radioactive substances (groundshine). The internal radiation exposure by inhalation is calculated as follows:

*DOSE_inh_* [*mSv*] = *Q_inh_* [*m*^−3 ^*s*^−1^]∙*DCF_inh_* [*mSvBq*^−1^]∙*TIC* (*x*, *y*) [*Bqsm*^−3^] (5)

DOSE_inh_ : Effective dose by respiration; Q_inh_ : Respiratory rate (3.34E−4 m^3^/sec); DCF_inh_ : Inhalation dose coefficient.

The external radiation exposure by a radiation plume can be expressed in a function on the concentration of atmospheric radioactive substances as shown below:

*DOSE_cl_* [*mSv*] = *DCF_cl_* [(*mSvs*^−1^) / (*Bqm*^−3^)]∙*TIC* (*x*, *y*) [*Bqsm*^−3^] (6)

DOSE_cl_ : Effective dose caused by cloudshine; DCF_cl_ : Dose coefficient on the cloudshine. 

The external radiation exposure caused by deposited radioactive substances is a function on the deposition concentration as shown below, and it should take in consideration the collapse rate according to the lapse of time:

*DOSE_gr_* [*mSvs*^−1^] = *DCF_gr_* [(*mSvhr*^−1^) / (*Bqm*^−2^)]∙*DEP* (*x*, *y*) [*Bqsm*^−2^] (7)

DOSE_gr_ : Effective dose caused by groundshine; DCF_gr_ : Dose coefficient on groundshine.

Therefore, the effective dose considering internal radiation exposure and external radiation exposure are as follows:

*ED* = *DOSE_inh_* + *DOSE_cl_* + *DOSE_gr_* (8)

The dose conversion factors used in this study were effective dose coefficients of ICRP60 and are shown in Table 1.

**Table 1:** Dose conversion factors for the estimation of exposure doses.

Isotope	Inhalation	Air Submersion	Surface Submersion
	(mSv Bq^−1^)	(mSv/Bqsm^−3^)	(mSv/Bqsm^−2^)
^137^Cs	3.90 × 10^−5^	9.28 × 10^−14^	2.99 × 10^−15^
^131^I	7.40 × 10^−6^	1.69 × 10^−11^	3.64 × 10^−13^

## 3. Results and Discussion

### 3.1. Atmospheric Dispersion Characteristics of Radioactive Substances

**Figure 1:**
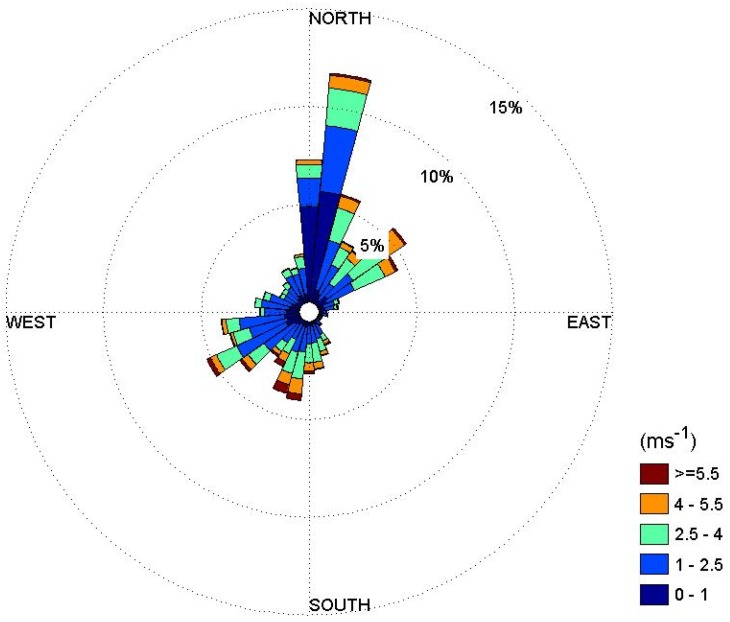
Annual wind-rose measured in Gwangju in 2011.

To estimate the concentration of radioactive substances in ambient air in the case of a nuclear accident, meteorological data are required. For an analysis of the atmospheric dispersion of radioactive substances, this study used the meteorological data measured by the Korea Meteorological Administration (KMA) in Gwangju near the site of the Yeoungkwang Nuclear Power plant. Figure 1 shows a wind-rose based on the wind direction and wind speed measured by KMA in 2011. 

It shows that northern and northeastern winds dominated overall, and the wind toward Gwangju, a major city, was not strong. To analyze atmospheric dispersion characteristics of radioactive substances using the Gaussian Plume Model, horizontal and vertical dispersion coefficients are required. Vertical and horizontal dispersion coefficients are shown as a function of atmospheric stability.

**Figure 2:**
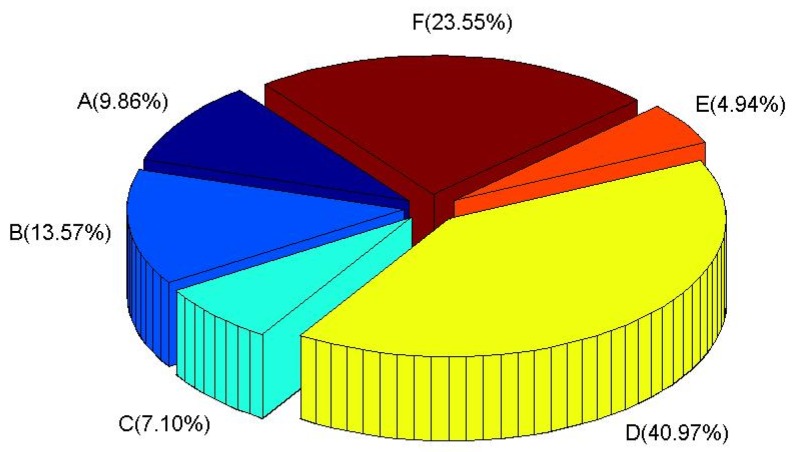
Distributions of atmospheric stability.

Figure 2 shows the atmospheric stability distribution estimated based on the meteorological data in Gwangju City. Atmospheric stability class is the index to explain the turbulence of the air parcel. A means very unstable which makes dilution of radioactive materials very well, whereas F means very stable which makes dilution of radioactive materials poor. The unstable conditions, A and B, which are advantageous to the dispersion of radioactive substances represent about 23% while E and F, which are disadvantageous to atmospheric dispersion take about 30%. D, which represents the neutral state of atmospheric stability occupies 40% of the total.

**Figure 3:**
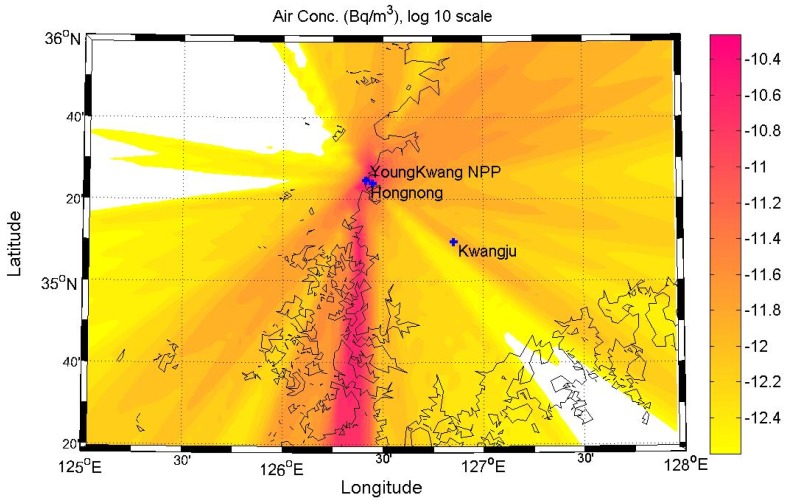
Hourly averaged air concentrations for a radioactive material.

**Figure 4:**
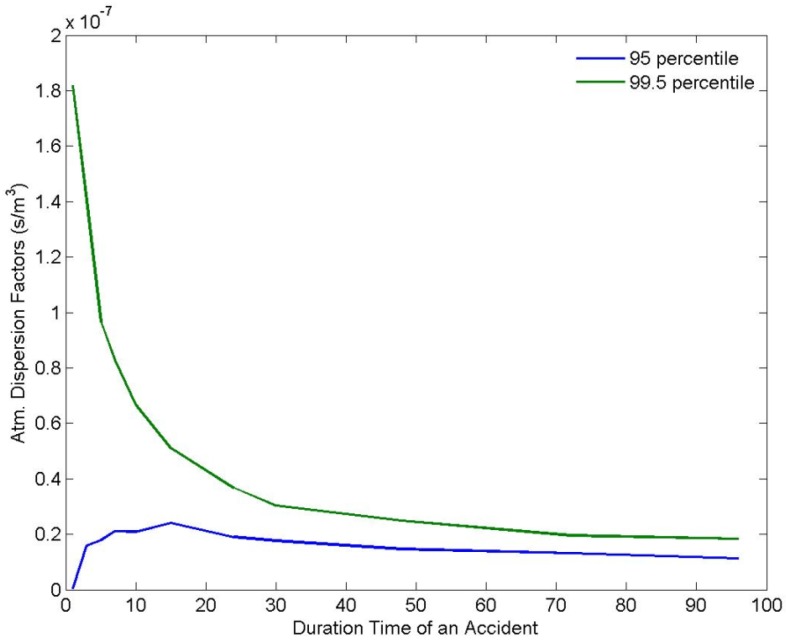
Variations of atmospheric dispersion factors by the duration time of radioactive effluents in the highly populated area.

Figure 3 shows the average atmospheric concentration of radioactive substances when the radioactive substances in 1 unit mass (1 Bq/sec) are released in air at a height of 50 m, in order to quantify the atmospheric dispersion characteristics around the Yeoungkwang Nuclear Power plant. As shown in the wind-rose in Figure 1, as the northern and northeastern winds are dominant, there are relatively higher concentrations of radioactive substances distributed in the ambient air in the southern direction of the nuclear power plant site than in the other directions. Figure 4 indicates atmospheric dispersion factors in Hongnong, a densely populated area near the power plant, according to the duration time, to quantify the atmospheric dispersion characteristics of radioactive substances. It was confirmed that as the length of emission duration increases, the 99.5th percentile of atmospheric dispersion factors decreased sharply. It is suspected that as the length of emission duration increases, the changes in wind direction increase as well, which facilitates the dilution of radioactive substances. 

In the event that the same quantity of radioactive substances is released, if the emission duration becomes longer than 7 h, the concentration in air decreased by about 50% compared to the case when the emission duration was one hour. Supposing the same amount of radioactive substance emitted, in the event that the emission duration is more than four days, the concentration of radioactive substances in air decreases by more than 90% compared to the case when the emission duration was one hour. When the emission duration is one hour or three hours, the 95th percentile of atmospheric dispersion factors showed a smaller value than the 5-hour duration time, in spite of the general assumption that it normally decreases when the length of the emission duration increases owing to the change in wind direction. As for the one-hour and three-hour durations, the atmospheric dispersion factors were overly distributed in the left side, which is assumed to be the main reason for the smaller value. 

### 3.2. Radiation Dose and Acute Health Effects on the Residents.

The Yeoungkwang Nuclear Power plant is located on the west coast of the Korean peninsula and six nuclear reactors are currently in operation. Because the type of nuclear reactors in the Yeoungkwang Nuclear Power plant are different from that of the nuclear reactors in the Fukushima Daiichi Nuclear Power plant in Japan, there is rare chance of the same kind of accident occurring. We estimated the radiation dose and health effects of the residents in the Hongnong area, a densely populated region near the Yeongkwang Nuclear Power plant hypothesizing that the source term, which is equivalent to the accident of Fukushima Daiichi Nuclear Power Plant, is released in the Yeoungkwang Nuclear Power plant. Japan Atomic Energy Commission (JAEC) reported Cs-137 1.2E + 16 Bq, I-131 1.5E + 17 Bq as the activity of radioactive substances released for about 10 days since March 11th, the accident date of Fukushima Daiichi nuclear Power Plant [11,12,13]. This study supposed the effective altitude of a radiation plume as 50 meters. In the Fukushima Daiichi Nuclear Power Plant accident, the emission of radioactive substances lasted for more than 10 days. This study estimated the radiation dose and acute health effects of the residents in Hongnong, a densely populated area in the event that the emission duration is changed from one hour to 96 hours.

**Figure 5:**
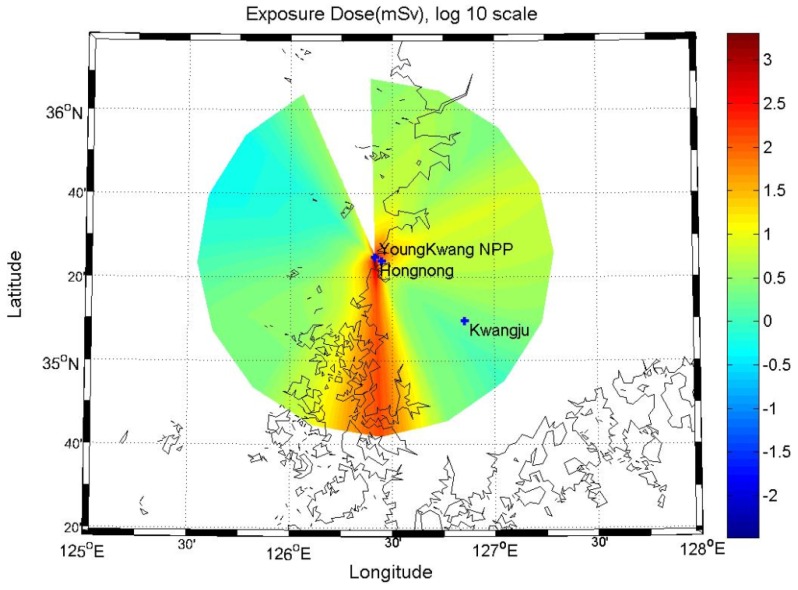
Hourly averaged effective radiation doses.

Figure 5 shows the estimated radiation dose within a radius of 80 km from the Yeoungkwang Nuclear Power plant in the event of a hypothetical nuclear accident, by using atmospheric dispersion factors that reflected the annual average atmospheric dispersion characteristics. The distribution of the radiation dose showed a similar pattern as the concentration distribution of atmospheric radioactive substances in Figure 3. As the northern and northeastern winds are dominating overall, it was confirmed that the there was a high radiation dose in the southern part of the nuclear site.

There is scarce possibility of acute health effects in a highly populated area as shown in Table 2 when the accident duration is 96 h. Table 3 shows the radiation dose according to the duration time of emission. In the event that the source terms of the accidents are the same, it was confirmed that as the emission duration gets longer, the radiation dose in a highly populated area decreases because the dilution of radioactive substances is facilitated smoothly. The 99.5th percentile of radiation dose for 96 h means that the value over the radiation dose will not occur statistically, except for 0.05%. That is, the exceedance probability is 0.05%. When the duration time of the accident is 96 h, the 99.5th percentile of the radiation dose does not exceed 10 mSv even without protective actions such as evacuation and sheltering.

**Figure 6:**
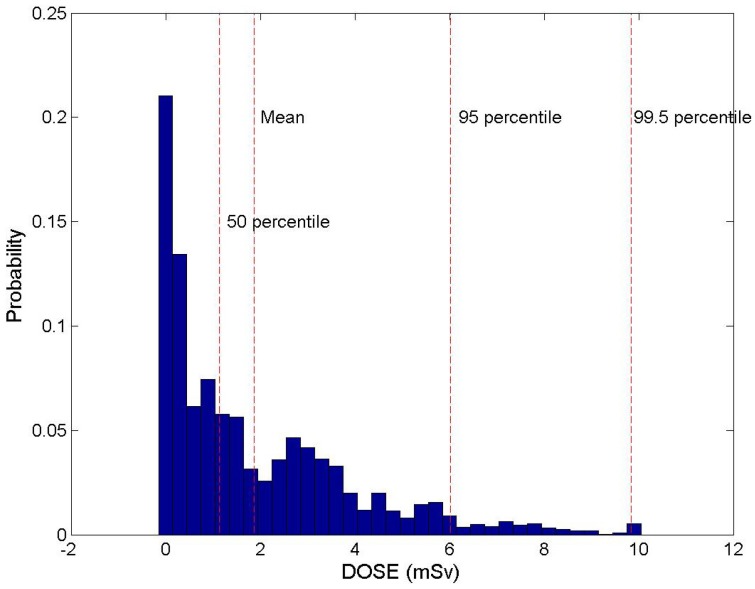
Frequency distribution of hourly radiation doses.

Figure 6 shows the frequency distribution of the radiation dose in Hongnong, a highly populated region, when the duration time of an accident is 96 hours. Radiation dose shows the gamma-type distribution and 1 mSv, which is the limit of radiation dose for an average person located around the 50th percentile. This means that if the accident happens in a certain period, the probability that the radiation dose does not exceed 1 mSv is about 50% even without any specific protective actions in the highly populated area. 

**Table 2:** Effective dose at Hongnong in case of nuclear accident at the YeoungKwang nuclear power plant (unit: mSv).

	Inhalation	Air submersion	Ground surface	Total
^137^Cs	5.70 × 10 ^−1^	4.06 × 10^−6^	1.31 × 10^−1^	5.70 × 10^−1^
^131^I	1.29 × 10^+^^0^	8.81 × 10^−3^	1.90 × 10^−7^	1.30 × 10^+^^0^
Total	1.86 × 10^+^^0^	8.81 × 10^−3^	1.90 × 10^−7^	1.87 × 10^+^^0^

The occurrence of an acute disorder after a nuclear accident can be estimated in the following Sigmoid function by using the sum that adds the radiation dose for 7 days after the accident to half of the radiation dose from the 7th day to the 23rd day:

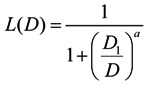
(9)

Here, D is a short-term radiation dose; D1 is the 50% lethal dose (LD_50_) of radiation; and *a* is a coefficient. In the case of a light acute disorder, serious acute disorder, and acute death, *a* is used as 7.6377, 9.8467, and 5.4190, respectively [14].

As we confirmed in the nuclear accident in Japan, massive amounts of radioactive substances had been emitted for more than 10 days. Therefore, an acute radiation exposure that may definitely affect health did not occur in a highly populated area, and no deaths by acute radiation exposure were reported. Supposing the emission duration as 96 hours, the atmospheric dispersion factor was 3.47E−9 s/m^3^. The radiation dose estimated in a highly populated area using this value was 1.87 mSv, of which 70% was caused by I-131, and the remaining 30% was caused by Cs-137 (Table 2). In terms of exposure pathways, radiation exposure by inhalation was 99.5% and radiation exposure by radioactive cloud and deposition was approximately 0.5%. However, because the radiation exposure caused by inhalation and a radioactive cloud does not occur after the emission of radioactive substances is completed, radiation exposure occurs only by deposited radioactive substances. In this study, exposure pathways of foods and drinking water are not considered, but in the radiological consequence evaluation for long-term phase these pathways should be considered. Furthermore, the exposure of radiation, especially I-131, from drinking water can be also important even for short-term periods.

**Table 3:** Radiation dose and acute health impact by effluent duration time.

Time	95 Percentile Dose (mSv)	99.5 Percentile dose (mSv)	Light Acute Disorder (/Person)	Serious Acute Disorder (/Person)	Acute Death (/Person)
1 h	2.24 × 10^−1^	9.77 × 10^+1^	1.74 × 10^−7^	1.23 × 10^−13^	1.84 × 10^−9^
3 h	8.48 × 10^+^^0^	7.55 × 10^+1^	2.42 × 10^−8^	9.71 × 10^−15^	4.54 × 10^−1^^0^
5 h	8.48 × 10^+^^0^	5.20 × 10^+1^	1.40 × 10^−9^	2.47 × 10^−16^	6.02 × 10^−11^
7 h	1.13 × 10^+1^	4.45 × 10^+1^	4.27 × 10^−1^	5.33 × 10^−17^	2.59 × 10^−11^
10 h	1.12 × 10^+1^	3.58 × 10^+1^	8.11 × 10^−11^	6.26 × 10^−18^	7.96 × 10^−12^
15 h	1.30 × 10^+1^	2.74 × 10^+1^	1.05 × 10^−11^	4.50 × 10^−19^	1.87 × 10^−12^
24 h	1.02 × 10^+1^	1.97 × 10^+1^	8.47 × 10^−13^	1.75 × 10^−2^	3.13 × 10^−13^
30 h	9.49 × 10^+^^0^	1.63 × 10^+1^	1.99 × 10^−13^	2.70 × 10^−21^	1.12 × 10^−13^
48 h	7.85 × 10^+^^0^	1.34 × 10^+1^	4.46 × 10^−14^	3.93 × 10^−22^	3.87 × 10^−14^
72 h	7.05 × 10^+^^0^	1.05 × 10^+1^	6.93 × 10^−15^	3.56 × 10^−23^	1.03 × 10^−14^
96 h	6.02 × 10^+^^0^	9.84 × 10^+^^0^	4.22 × 10^−15^	1.88 × 10^−23^	7.27 × 10^−15^

## 4. Conclusions

This study aims to quantify the effect of duration time of a nuclear accident on the radiation dose of a densely populated area and the acute health effects. The important results obtained from this research are as follows:

The increase in length of emission duration can greatly reduce the concentration of radioactive substances in ambient air by dilution action, when a severe accident occurred.

In the event of an accident in the Yeoungkwang Nuclear Power plant in Korea, if the emission lasts for more than 4 days, the concentration of the radioactive substances in the ambient air in a highly populated area decreases by more than 90% compared to when the duration is one hour.

As for a hypothetical accident in the Nuclear Power plant in Korea, acute health effects on the human body in a highly populated area decrease sharply according to the increase in the length of emission duration due to the dilution actions of radioactive materials. Therefore, it is judged that the proper control of the emission time of a nuclear accident can minimize the health effects on residents. 
